# A topological map of the genetic components of grapevine—Admixture meets SOMmelier machine learning

**DOI:** 10.1371/journal.pcbi.1013882

**Published:** 2026-02-20

**Authors:** Anush Baloyan, Tomas Konecny, Emma Hovhannisyan, Nate Zadirako, Maria Nikoghosyan, Hans Binder

**Affiliations:** 1 Armenian Bioinformatics Institute (ABI), Yerevan, Armenia; 2 Institute of Molecular Biology (IMB) of the National Academy of Sciences of the Republic of Armenia, Yerevan, Armenia; 3 Interdisciplinary Centre for Bioinformatics (IZBI), Universität Leipzig, Leipzig, Germany; University of Pittsburgh, UNITED STATES OF AMERICA

## Abstract

Inferring the genetic structure at the subpopulation level is crucial for understanding the demographic histories that shape genetic diversity. Among the most widely used approaches are methods based on admixture and structure modeling—named after the respective software tools—which have become standard due to their intuitive, interpretable outputs. In this study, we address a key methodological question: how does the traditional admixture-based decomposition of genetic components in multilocus population data relate to clustering approaches that leverage machine learning, specifically Self-Organizing Maps (SOMs)? We implemented this approach through our custom SOM-based tool, SOMmelier, which enables the portrayal of genetic structure by identifying modules of co-mutated SNPs and arranging them in a topology-aware genetic landscape. Topology-awareness refers to the organization of genetic modules in a two-dimensional map, where their spatial proximity reflects mutual similarity. We applied Admixture and SOMmelier to investigate the population genetics of European grapevine. Based on prior literature, we considered up to six genetic components, which formed a genetic landscape that closely mirrors the geographic expanse of the classical Mediterranean world—from Western Asia through the Caucasus to Western Europe. The resulting topology reflects the dynamic spatial and temporal nature of grapevine domestication and diffusion. We demonstrate that SOMmelier can recover the genetic components identified by Admixture solely through statistical clustering. By integrating the topological structure of SNP co-variation, it offers perspectives on population structure, evolutionary history, and trait associations in grapevine—and has applicability to other species and systems in population genetics.

## Author summary

Populations are shaped by both evolutionary processes and human activities such as breeding, which is especially evident in cultivated animals and plants. The genetic variation within these populations is encoded in their genomes, and can often be described as a combination of distinct genetic “admixture” components using standard computational approaches. In this study, we ask: How does this admixture-based view of population structure compare to the representation provided by machine-learning–based Self-Organizing Maps (SOMs)? SOMs offer an intuitive way to explore complex molecular data and reveal relationships that might be missed by conventional methods. Using cultivated grapevine as a model—an economically important, globally distributed crop with a long history of domestication—we show that our SOMmelier approach not only recapitulates known genomic components but also constructs a topology-aware genetic landscape. This landscape reflects the geographic distribution of grapevine accessions across Europe and West Asia, and preserves genetic footprints of cultivation history spanning the past 11,000 years. Importantly, SOMmelier both complements and extends genetic admixture analysis, highlighting its potential for broad application in population genetics beyond grapevine.

## Introduction

Inferring the genetic structure of populations at the subpopulation level from genotype data is essential for understanding the evolutionary forces and demographic history shaping these populations. In a general context, such analyses play a critical role in conservation biology, population management, and in controlling for population stratification in genome-wide association studies (GWAS) of complex traits, including heritable diseases (see [1] for a review). Among the most widely used methods are those based on structure and admixture modeling, named after the software tools STRUCTURE [2] and ADMIXTURE [3]. These methods, along with their numerous extensions and variants [1,4–7] have become standards in population genetics due to their intuitive and interpretable outputs. They rely on statistical models that assume Hardy–Weinberg within ancestral populations and linkage disequilibrium between loci. These algorithms estimate individual admixture proportions (ancestries), denoted as Q, for each of N sampled individuals, across K hypothesized source populations [3]. Results are typically visualized as stacked bar plots representing the K × N matrix of ancestry proportions.

Model-based admixture inference is often complemented by sample similarity analyses that employ non-model-based statistical methods such as Principal Component Analysis (PCA) [8,9], k-means clustering [10], or Uniform Manifold Approximation and Projection (UMAP) [11]. These approaches do not assume specific population genetic models but instead rely on general statistical principles, such as minimizing within-cluster genetic variance while maximizing between-cluster variance. For example, PCA reduces high-dimensional genotype data into a few orthogonal principal components (PCs), facilitating visualization of population structure. Compared to model-based methods, these non-parametric approaches require fewer assumptions, are computationally efficient, and scale well to large datasets. However, they lack an explicit evolutionary model, making biological interpretation less straightforward. Moreover, PCA outcomes may be confounded by demographic history or uneven sampling designs [12,13].

Importantly, model-based and non-model-based approaches are complementary. Admixture analyses provide insights into the individual-level genetic composition, while sample similarity methods visualize how these individual ancestries manifest in overall population structure. This dual approach has been employed extensively and constantly in nearly all areas of population genetics. A few illustrative examples refer to studies of human population history, including investigations into the origin of Indo-European languages [14,15], and the domestication of animals such as horses [16], dogs [17], and cats [18]. Similarly, in plant domestication studies, the same methodological framework has been applied to crops like tomato [19], rice [20] or Persian walnut [21]. Despite the diversity of research contexts, these representative studies exemplify the power of combining admixture modeling and PCA clustering to unravel phylogenetic relationships and trace the spatial and temporal dynamics of subpopulation divergence.

We recently developed a method called Self-Organizing Maps (SOM) portrayal, which performs dimensionality reduction and clustering of genetic features based on their *Euclidean* distance similarities across samples [22,23]. This approach provides a model-free, topology-preserving visualization of the covariance landscape of high-dimensional data, enabling both intuitive single-sample resolution and extensive knowledge-mining. The method has been called “portrayal” because of its unique property to generate an individual SOM image for each sample, which visualizes the specifics of its data landscape. The resulting molecular-genetic portraits allow for the identification of co-regulated feature modules, typically representing functional gene sets [24]. SOM portrayal has been successfully applied to various omics data types, including gene expression, DNA methylation, and copy number variation, across diverse disease contexts [25–27]. Its application to single-nucleotide polymorphism (SNP) data has enabled novel insights into SARS-CoV-2 viral evolution [28], disease incidence prediction using polygenic markers [29], and the reconstruction of human migration trajectories out of Africa based on data from the 1000 Genomes Project [30].

In these studies, we observed that the genetic clusters derived from the SOM analysis appear to correspond to admixture components previously reported in the literature. This observation led us to hypothesize that SOM-extracted clusters may correlate with these admixture components. In this publication, we therefore investigate the relationship between genetic components identified through admixture analysis and the covariance landscape generated by SOM. More specifically, we aim to determine whether SOM can be used to construct a topology-aware representation of genetic components which, in addition to performing the genetic decomposition achieved by admixture analysis, also provides information on the spatial relationships among components and visualizes them in an interpretable manner.

We selected the cultivated grapevine as an example to prove our hypothesis. Previously we analyzed a comprehensive SNP dataset comprising nearly 800 grapevine cultivars sampled globally using a SOM application called “SOMmelier” [31,32]. This analysis focused on the geographic structuring of accessions and their assignment to distinct periods in the domestication and historical cultivation of grapevine [33,34], however without considering the intrinsic genetic structure of the data.

Grapevine genetics is interesting because the Eurasian grapevine (Vitis vinifera) exhibits great morphological and genetic diversity with thousands of varieties described in historic and contemporaneous records. This great diversity is deeply rooted in early viticulture. The domestication and spreading of grapevine as well as the gene flow history had been described in many studies to assess the population genetic diversity, structure, and relatedness, and to infer the most likely migration events throughout Europe from Caucasus around the Mediterranean Basin [33,35–38]. Moreover, vitis vinifera is one of the most widely cultivated plant species of agricultural interest, and is extensively appreciated for its fruits and the wines. Considering the high socio-economic impact of the wine sector all over the world, in recent years, there has been an increase in work aiming to investigate the biodiversity of grapevine germplasm available for breeding programs [39].

In this publication we demonstrate that SOMmelier can recover the genetic components identified by Admixture solely through statistical clustering. From the perspective of grapevine genetics, we interpret this landscape in terms of the geographic dissemination of genetic components from “Iberia to Iberia”, i.e., from the historical Kingdom of Iberia in the South Caucasus (encompassing parts of present-day Georgia and Armenia) in the east, to the Iberian Peninsula in the west. Within this framework, we explore associations with traits as revealed by SOMmelier and address the selection of genetic markers representative of specific genetic components. SOMmelier thus provides a complementary approach that bridges model-based Admixture analysis and model-free PCA clustering. It enhances genetic data analysis by incorporating the topology of intrinsic SNP co-variation, offering novel insights into population structure, evolutionary history, and trait association of grapevine.

## Results

### Admixture and SOM portrayal: Visualizing genetic diversity across geographic regions

**Fig 1:**
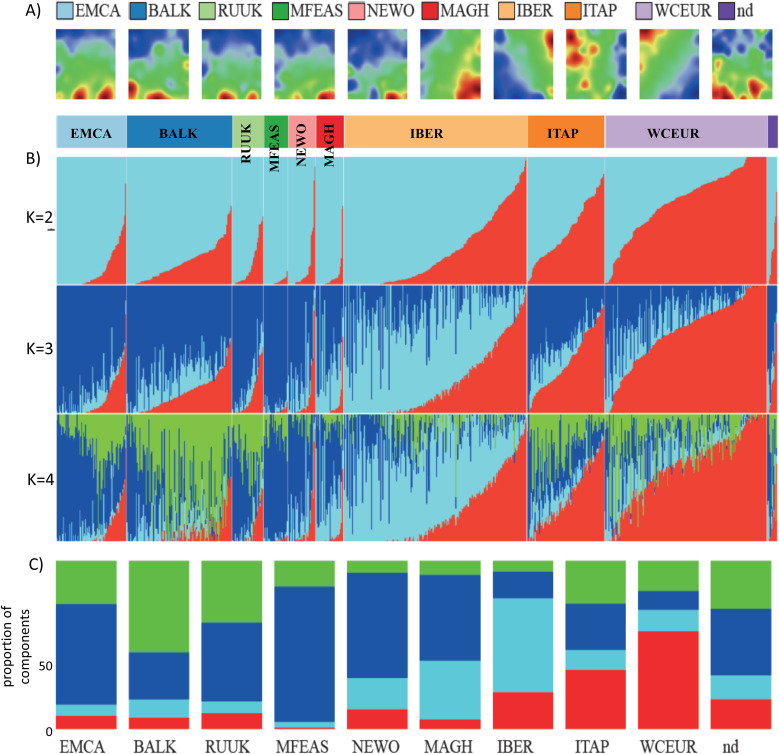
SOM portrayal and admixture analysis of 783 vine accessions selected from nine geographic regions around the world. **A)** Mean SOM portraits were calculated over all individual portraits from the respective region. The red/blue “spots” cluster co-mutated SNPs with high/low SNP-score values in the respective region. They provide topology-preserving images which “portray” the SNP landscapes for each of the geographic regions. **B)** ADMIXTURE results for K = 2-4 genetic components were arranged by geographic groups. The color bar indicates the geographical region for each sample in the admixture plot. **C)** Proportion of the four genetic components (K = 4) across the geographic regions indicates dominance of the blue component in MFEAS, the maximum fraction of the red component in WCEUR, of light blue in IBER and of green in BALK. Vine accessions without geographic specification were marked as “nd” (not determined).

In the initial step, we conducted admixture and self-organizing map (SOM) portrayal analyses on 783 vine accessions from the Laucou dataset [31], organizing them according to nine geographic regions (Figs 1A, 1B and S1): Eastern Mediterranean and Caucasus (EMCA), the Balkans (BALK), Russia and Ukraine (RUUK), Middle and Far East (MFEAS), New World (NEWO), Maghreb (MAGH), Iberian Peninsula (IBER), Italian Peninsula (ITAP) and Western and Central Europe (WCEUR). The SOM images depict genetic landscapes, with clusters of co-mutated single-nucleotide polymorphisms (SNPs) visualized as spot-like regions. SNPs with high values of the excess minor allele frequency (eMAF) hereafter referred to as the SNP-score (see [32] and materials and methods section) are highlighted in red, while regions with low and intermediate SNP-score are marked in blue and green, respectively. The colored areas mainly refer to the minor (red), major (blue) and heterozygous (green) alleles, respectively. A preliminary examination of the SOM portraits reveals distinctive red and blue spot patterns for each geographic region, characterized by both overlapping and unique regions, which indicate shared and region-specific SNP patterns. For instance, red spots are predominantly located in the lower portion of the SOM portraits for EMCA, BALK, and MFEAS, whereas in IBER, ITAP, and WCEUR, the red spots are concentrated in the upper regions. Overall, we identified approximately seven to ten such spot-like clusters of co-mutated SNPs across the different geographic regions. Visual inspection and comparison of the SOM images revealed similarities of the spot patterns, e.g., of EMCA, RUUK and MEFAS regions and clear differences between EMCA and IBER, ITAP and WCEUR regions while, e.g., EMCA compared with MAGH and BALK showed partial similarities. Hence, the SOM portraits enable visual estimation of the degree of genetic similarities of the accessions originating from different geographic regions.

**Fig 2:**
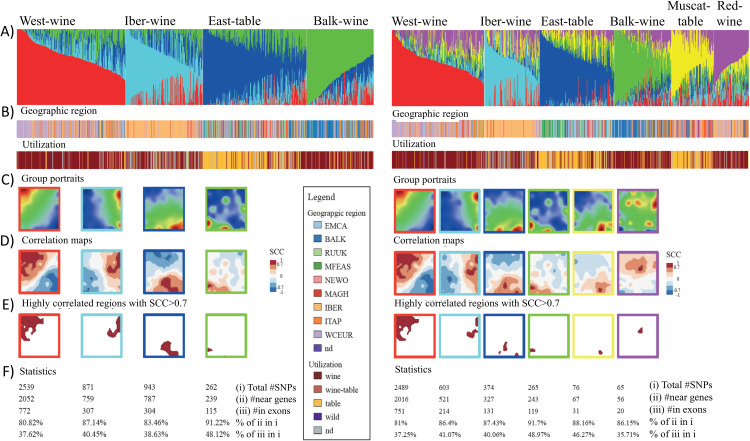
Admixture-SOM correlation analysis for K = 4 (left part) and K = 6 (right part). A) Genetic components were sorted by decaying maximum component. A-B) “Nicknames” of the groups were chosen according to their major geographic origin and utilization. The two additional components for K = 6 compared with K = 4 accumulate accessions with Muscat flavor table vine and wine-usage accessions with red berry skin color. C) Mean SOM portraits of each admixture component, which shows characteristic spot patterns correlating with the respective admixture component. D) The correlation maps visualize the Spearman Correlation Coefficients (SCC) between the admixture components and the meta-SNP profiles of the SOM. Regions of large SCC virtually agree with the red spots of high SNP-score values in the SOM portraits. E) Map of high SCC > 0.7 select SNPs highly correlating with the respective genetic component. F) SNP-statistics in the high-SCC regions. Lists of SNPs and genes in the correlation areas are provided in S1 (K = 4) and S2 Tables (K = 6). Overall, this figure clearly reveals the relation between the genetic components generated by admixture analysis and the spot patterns obtained by SOM.

Admixture analysis assuming two genetic components (K = 2), represented in light blue and red, suggests a relationship between the red spot patterns in the SOM portraits and the amplitude of the admixture components (Fig 2A and 2B). However, the admixture plots also reveal a broad distribution, indicating significant genetic heterogeneity. When a third genetic component (K = 3) is introduced, a distinct diversification is observed, particularly in the genetic composition of IBER accessions (light blue). The addition of a fourth component (K = 4) highlights further differentiation, most prominently in the BALK region and, to a lesser extent, in ITAP (green). To provide an overview, we calculated the mean genetic composition for each geographic region (Fig 2C). The dominant ancestry components are group-specific: WCEUR is enriched for the red component, MFEAS for blue, IBER for light blue, and BALK for green. These four major components identified in the admixture analysis correspond to specific regions in the SOM portraits, such as the upper left and upper right corners for WCEUR and IBER, respectively, and the lower edge of the map for EMCA/MFEAS and BALK as mentioned above. On the other hand, the genetic components distribute over the geographic regions, reflecting their genetic heterogeneity. Hence, these observations indicate that the degree of similarity between the SOM portraits from different regions associates with the genetic components extracted from admixture analysis.

In summary, the comparison of SOM portraits and admixture components across nine geographic regions reveals parallel patterns in the intrinsic SNP covariance structure. This analysis suggests the presence of two major clusters at a coarse level of resolution, and at least four clusters at a higher level of granularity.

### Admixture components associate with SOM spot patterns

Next, we assigned the four leading genetic components as archetypes, considering their main geographic location and grape utilization in agreement with the classification proposed in [31]. These archetypes were defined as West-wine (western wine grapes), IBER-wine (Iberian wine grapes), East-table (eastern table grapes) and BALK-wine (Balkan wine grapes, Fig 2A and 2B, left part, full gallery of individual cultivar portraits is shown in S1 Fig). Table grape utilization was predominantly associated with the blue-colored East-table group, comprising accessions primarily from RUUK, MFEAS, and EMCA. In contrast, wine grape utilization was enriched in accessions associated with WCEUR (red component), BALK (light blue), and IBER (green).

The mean SOM portraits, averaged for each archetype, revealed specific and virtually non-overlapping regions for the respective groups (Fig 2C). To link the SOM spot patterns with genetic components, we calculated Spearman rank correlations (SCC) between component-percentage profiles (Q-values) and the mean SNP-score profiles for each SOM pixel. The resulting correlation maps, which depict positive correlations in red and negative correlations in blue, provide a “spatial” representation of the genetic components in the SOM showing strong similarities with the red spots in the mean SOM of the respective component (Fig 2D, compare with 2C). Hence, each archetypical component was associated with a distinct region in the SOM.

SNPs linked to each genetic component exhibited high SNP-score values in their respective SOM regions, enabling the extraction of explicit SNP lists based on a Spearman correlation threshold of SCC > 0.7 (Fig 2E). The number of SNPs identified within these regions varied by over an order of magnitude, ranging from 2,539 (red cluster, associated with West-wine accessions) to only 262 (green cluster, associated with BALK-wine accessions). Of these mutations, 78–84% were found near genes (Fig 2F).

We repeated this analysis for six admixture components (K = 6, Fig 2, right part) which provided two additional components called Muscat-table (yellow) and Red-wine (violet) (see below and Fig 2A and 2B; right part). The former component can be regarded as a derivative of the East-table group while the latter one arises mainly from the Iber-wine and partly former one is virtually a “spin-off the West-wine groups, as revealed by their SOM portraits resembling those of their “parent” groups (Fig 2C; right part). On the other hand, all groups are characterized by their specific spot patterns in the mean SOM portraits as well as in their correlation maps (Fig 2C-2E; right part). As for K = 4, the number of genes associated with the correlation spots progressively decreases from the left to the right (Fig 2F; right part).

In summary, correlation analysis between admixture components and SOM-profiles provides correlation maps where regions of high correlation virtually agree with regions of high SNP-score. These distinct SOM areas provide associated genetic features such as SNP- and gene-lists for further downstream analysis.

### Resolving admixture components in the SOM portraits

**Fig 3:**
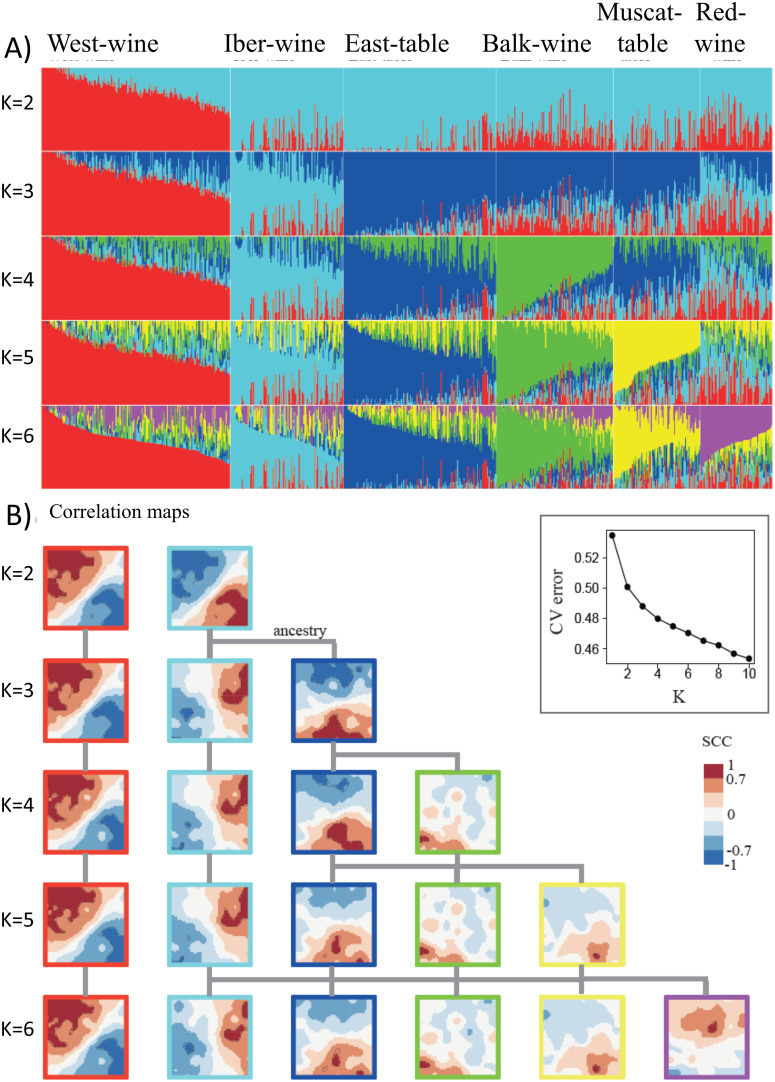
Admixture-SOM correlation analysis for K = 2-6. (A) Admixture plots and (B) the respective correlation maps showing hierarchical ancestry of spot patterns, as indicated by the grey lines. Each additional genetic component virtually splits a previous spot region thus enabling to construct ancestry relations. The CV-error plot monotonously decays with the number of components considered thus suggesting that K > 6 could be an option for identifying more components (see discussion).

Next, we ask about associations between the admixture genetic compounds and the SOM-landscape in a more systematic way for K = 2–6 (Fig 3). Two-component clustering (K = 2) correlates with SNPs in the upper left (red component) and lower right (cyan) of the SOM, thus dividing the map along the diagonal, mostly into wine-grapes from the west and table grapes from the east (compare Fig 3A and 3B). Further increasing K from 3 to 4 progressively decomposes the cyan component and the correlation spots in the SOM at the right and virtually leaves the red component and the left spot unchanged. Increasing to K = 5 (yellow) decomposes the blue component and further increasing to K = 6 (violet) decomposes the red and cyan components, thus overall giving rise to a hierarchical split of admixture components that are visualized by their correlation spots in the SOM. Notably, K = 5 and 6 provide well-located areas in the SOM referring to the yellow and violet admixture components, respectively. The CV-error plot did not reach a minimum thus suggesting further fine-granular components for K > 6.

However, the incremental change of the CV-error between subsequent k progressively decreases for k < 5 and then converges to a constant decay suggesting that there are about four main ancestral groups while k > 4 reflect more subtle substructures resulting possibly from complex evolutionary dynamics not accessible by clear-cut clustering as revealed by the continuous changes of the amplitudes of the maximum genetic components for virtually all k which vary continuously between more than 90% to less than 50%. Importantly, a recent, independent whole genome sequencing study of more than 2,000 cultivated and wild vine accessions of an analogous geographic distribution as used here delivered a very similar course of the CV-error for admixture analysis (see S8B Fig in [33]), i.e., a steep decay for k < 5 and a constant one for k > 4, which in consequence let the authors cut the number of relevant clusters for cultivated vine to K = 6 in agreement with our cut-off. We will address this issue below and argue that K = 6 is a suitable choice that reflects intrinsic genetic heterogeneity of the grapevine accessions without over-granularization in agreement with previous groupings of vine accessions from the same regions [31,33].

Overall, we find that two genetic components, K = 2, oversimplify diversity of grapevine as expected, however the contributions to West-wine (red) from the other components can be readily estimated. Four components provide a reasonable genetic stratification covering the major clusters, while K = 6 further refines them and extracts genetic associates to Muscat flavor, originating mainly from the East, and red berry skin color, originating mainly from the West. Correlation with the SOM landscape identifies spot-clusters containing SNPs of the genetic components, up to the maximum resolution of six components.

### Associations between genomic components and phenotypes

**Fig 4:**
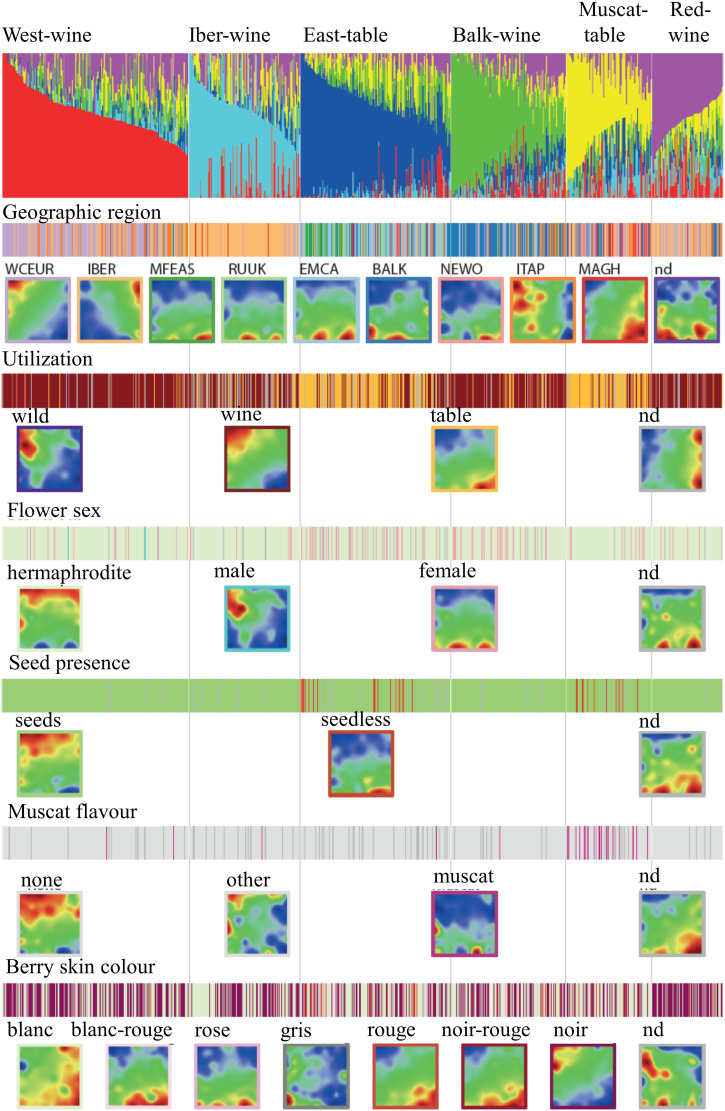
Phenotype association of the admixture components. The color bars illustrate the distribution of the respective accessions across the dataset. The SOM images represent mean portraits averaged over the accessions of the respective phenotype as assigned by the color frame around each portrait (sample sizes of the subgroups are given in the brackets). Comparison of the mean SOM portraits enables to establish mutual relatedness between them, e.g., that WCEUR accessions are dominated by wine utilization associated with seed presence while table usage dominates in seedless eastern vines. See S2 Fig for running averaged percentages of the phenotypes across the genetic components.

We next examined the distribution of accessions with various phenotypic characteristics across the K = 6 admixture stratification of grapevine cultivars, alongside their mean self-organizing map (SOM) portraits (Fig 4). Accessions from different geographic regions exhibited distinct enrichments in specific admixture components, which were reflected in their respective nicknames (e.g., “West Wine” (red) and “East Table” (blue)). An exception is the “Muscat Table” (yellow) component, which comprised accessions from both eastern and western regions and also included cultivars originating from Northern Africa (Maghreb) (S2 Fig). Notably, Italian accessions (ITAP) primarily contributed to neighboring regions, particularly WCEUR and BALK, forming a characteristic spot pattern linking that of WCEUR and BALK accessions. Table grape utilization was predominant in two of the six admixture components, although a significant proportion of wine and dual-purpose cultivars was also observed in the eastern regions (EMCA and MFEAS). In the SOM portraits, the wine-to-table usage gradient largely aligned with the west-to-east axis of the corresponding spot modules. Table grape utilization was associated with seedless and muscat flavor, both of which exhibited distinct spot patterns in the SOM portraits. Hence, phenotypic traits enriched in the different admixture components are associated with specific spots and the included SNPs which enable the intuitive perception of their mutual relations.

### The topology of the cultivar landscape

**Fig 5:**
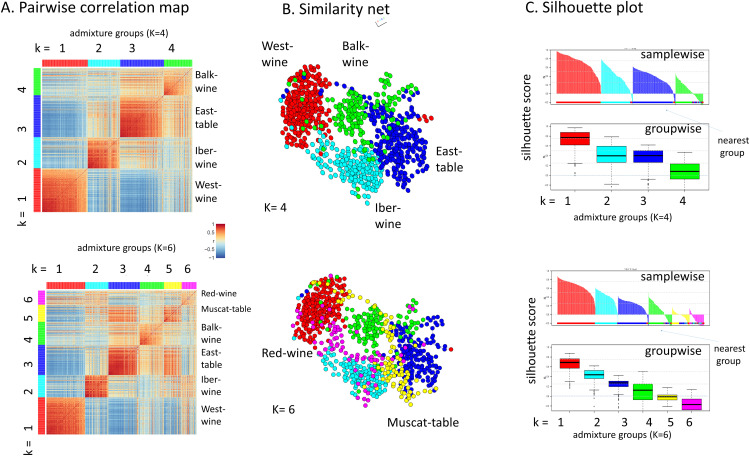
The topology of cultivar (sample) landscape: (A) Pairwise correlation maps of the individual SOM portraits sorted by admixture groups reveal intragroup similarities (brown squares along the diagonal) as well as intergroup similarities (brown off-diagonal regions). (B) Similarity net of the cultivars colored by their admixture groups for K = 4 and K = 6. The two additional components indicate accessions “bridging” the West-wine and East-table groups. (C) The silhouette score evaluates the “strength” of cluster membership with single (sample-wise) accession resolution and with group-wise resolution. The silhouette score below zero indicates preference for another cluster as indicated by the color bar. The robustness of the clusters progressively decreases with increasing k = 1 – 6 thus resembling the CV-error plot in Fig 3. For k = 5 and 6 the silhouette score becomes partly negative for considerable part of the accessions meaning that they are possibly close to overfitting.

Admixture analysis provides a highly informative composition plot of the genetic components considered, effectively projecting high-dimensional genetic data into a lower-dimensional space. However, this projection does not directly reflect topological relationships, i.e., the arrangement of data points within the data space. Pairwise correlation maps (PCM) of the SOM portraits visualize the covariance structure of the SNP data across accessions (Fig 5A). When samples are sorted according to K = 4, four distinct correlation clusters emerge (represented in brown squares along the diagonal). In a correlation similarity network representation, these clusters appear as four relatively discrete data clouds, each corresponding to a distinct genetic component (Fig 5B). A silhouette plot estimates the compactness and mutual overlap of these clusters. The positive silhouette values for the red, cyan, blue, and green components indicate a stronger association with their assigned cluster compared to the next most similar cluster (Fig 5C).

For K = 6, the PCM reveals additional off-diagonal brown regions, suggesting increased overlap between certain genetic groups. For example, overlap is observed between the blue (East table) and yellow (Muscat table) groups, as well as, to a lesser extent, between the cyan (Iber wine) and magenta (Red wine) groups. This pattern aligns with the mutual relationships observed in the SOM portraits (compare with Fig 3). In the similarity network representation, the additional components are positioned accordingly, with their respective group clusters connected and overlapping, leading to negative silhouette scores for most accessions meaning that k = 5 (yellow) and especially k = 6 (magenta) dominated samples exhibit partly stronger extra- than intra-cluster similarity scores. For example, magenta “red wine” (k = 6) accessions with negative silhouette score show mostly closer similarities with West wine (red) and Iber wine (cyan, see the bar plot below the silhouette score assigned as “nearest group” in Fig 3C) as visualized also by the overlap of the samples in the sample similarity net. Hence, the silhouette score suggests convergence towards a maximum number of genetic components of K = 4–6 supported by their visualization in the SOM but in apparent contradiction to the CV error plot (Fig 3, see discussion).

Notably, the topology of the data space indicates the greatest separation between the West wine and East table clusters, with the Iber wine and Balk wine groups positioned in between. This arrangement roughly corresponds to the geographic distribution of the respective regions of the accessions. The Muscat table and Red wine groups overlap with the East table group with Iber wine with West wine, respectively. Alternative similarity representations, such as phylogenetic trees or independent component analysis (ICA), largely preserve this topology but may alter distance relationships, offering alternative perspectives on data representation in sample space (S3 Fig). In summary, multidimensional mapping of the accessions’ SNPs SOM portraits provided data landscapes reflecting similarities between the sample data.

### The topology of the SNP landscape

**Fig 6:**
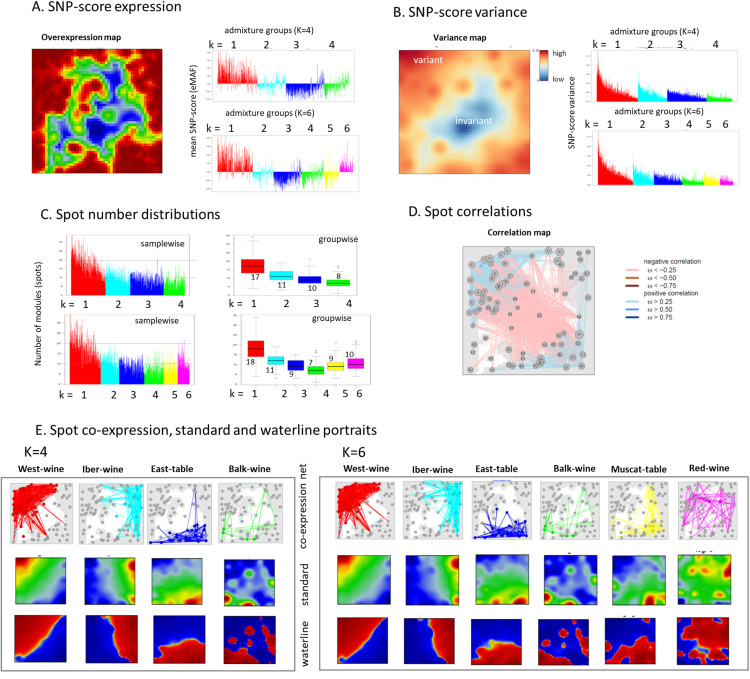
The topology of the SNP landscape. **(A)** The SNP-score overexpression map shows spot-like regions of high SNP-scores observed in the individual portraits. The mean SNP-score per sample stratified for K = 4 and K = 6 admixture components shows that the k = 1 (red) components refers to positive values while SNP-scores k = 2- 5 are virtually negative thus indicating a split of k = 1 versus the rest except k = 6 constituting an offspring of k = 1. Note that the SNP-score is centralized meaning that its mean over all accessions equals zero. **(B)** The SNP-score variance map indicates regions of high/low SNP-score in red/blue, respectively. The variance of the SNP-score per sample progressively decays with increasing **k. (C)** The number of spots detected per individual portraits decays for k = 1 – 4 but remains roughly constant for k = 3 -6. **(D)** The spot correlation map indicates positive correlations, usually between neighboring spots, as blue lines and negative correlations, usually between spots in opposite regions of the map. It indicates a correlation network dividing the genetic make-up of western-wine accessions from that of eastern table accessions. **(E)** Group portraits for K = 4 and K = 6. The spot co-expression maps show the network of co-expressed spots for each group, the standard portraits illustrate overexpressed SNP-score regions, and the waterline portraits depict regions with positive and negative SNP-scores in red and blue, respectively. Overall the topology of the SNP landscape is characterized by distinct groups of co-mutated SNP appearing as spot-like patterns where similar co-mutation patterns are located closely together while dissimilar (anti-correlated) patterns are located in distant areas of the map. Moreover, the genetic components k = 1- 6 refer to decaying variability and robustness.

As its unique property, SOM portrayal generates “individual” images of each accession, which enables visual comparison of their SNP landscapes (S1 Fig), which, in contrast to the cultivar landscape discussed above, visualize the differential SNP-scores in a SNP-centered coordinate system. The characteristic features of these portraits are the (red) “spot” modules representing clusters of co-mutated SNPs showing a high SNP-score in the respective accession (**S4**A Fig). Our SOM program generates a “personalized” summary map, providing an overview of all spot modules observed in the individual portraits (Fig 6A, left part). The spot modules cover virtually all the correlation areas discussed above (**S4**B Fig). For an overview, we also calculated the mean SNP-score of each accession (Fig 6A, right part). The variance of the meta-SNPs is shown in the variance map (Fig 6B). The SNP-score variance in each group decays with the fraction of the major component and is largest for the red-component but then levels off with increasing k (k = 1…K). The number-distribution of detected spots shows a similar behavior, which reflects the relation between metagene variance and spot detection (Fig 6C). Note that these variance measures decay with increasing K and converge for K > 4 thus associating with the decaying silhouette score revealing also a possible rationale for decay of the CV-error for K > 4, namely the intrinsic variance of the data which doesn’t allow to extract clear cut clusters (Fig 5C).

Next, we examined the correlation structure between the spot modules which anticorrelate across the diagonals of the map, especially between the red (k=1) West wine related spots and the blue (k=3) East table related spots (Fig 6B, red lines), while adjacent spots mainly positively correlate (blue lines), which agrees with the correlation structure of the cultivars shown in the PCMs (Fig 5A). For component-specific spot-networks, we calculated spot co-expression maps which connect spots appearing together in more than 50% of the individual portraits of each group and compared them with the mean group portraits (Fig 6E). Trivially, co-expressed spots accumulate in the region of mean spot overexpression but also reveal their diversity, particularly for k>3. Compared with the standard coloring, the waterline coloring better visualizes the area of SNP-scores greater and less than zero in red and blue, respectively.

Hence, the topology of the SNP landscape is characterized by the modular spot patterns, their correlation and co-expression networks, which divide it into areas of invariant SNPs and highly variant ones. Overall, the SNP landscape thus provides a network presentation with the spots as nodes and their co-expression links and/or mutual correlations as edges. It complements the cultivar landscape (see previous subsection) by providing a feature-centered perspective, which visualizes a “topology-aware” SNP-landscape visualizing proximity relations of the genetic features in the vine genomes.

### Spot segmentation, genetic markers and knowledge mining

**Fig 7:**
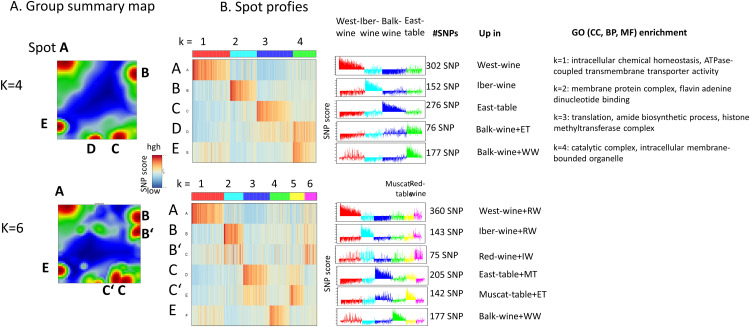
Spot module analysis of the mean group portraits for K = 4 and K = 6 (see also Fig 6E). (A) The group summary maps provide an overview of the spot modules of the group portraits labelled with uppercase letters. (B) SNP-score profiles reveal the group-specific upregulation of the SNP-score. Gene ontology (GO) enrichment analysis considers three categories: cellular component (CC), biological process (BP) and molecular function (MF). Lists of SNPs in the spots are provided in S3 (K = 4) and S4 Tables (K = 6). Enrichment analysis of functional themes is provided in S5 (K = 4) and S6 Tables (K = 6). Hence, segmentation of the spot modules enables to identify and to extract characteristic SNPs referring to the different genetic components.

Next, we aimed to identify genetic features—specifically SNPs and their associated genes—linked to the genetic components inferred by Admixture analysis. To achieve this, we generated group summary maps that provide an overview of the mutation “spots” detected in the group portraits for K = 4 and K = 6 (Fig 7A). The overall spot modularization is largely conserved between K = 4 and K = 6 (spots A–E), although additional resolution is observed at K = 6, where “double spots” B′ and C′ emerge alongside B and C. These reflect a subdivision of features between the cyan (K = 2, Iberian wine) and magenta (K = 6, red wine) groups for spots B and B′, and between the blue (K = 3, Eastern table grape) and yellow (K = 5, Muscat table grape) groups for spots D and D′. These patterns are indicative of admixture events among the respective genetic groups.

The SNP-score profiles of these modules closely mirror the admixture component proportions (compare Figs 7B and 2A), suggesting a strong correspondence between genotype composition and the detected population structure. Each spot module contains between 76 and 360 SNPs, which are mapped to a nearly equivalent number of genes. Gene set enrichment analysis using Gene Ontology (GO) terms revealed that the genes within each spot module are associated with distinct functional categories (Fig 7B, right panel), providing insights into the biological relevance of the genetic components.

For example, k = 1 (red, spot A) is associated with enhanced mitochondrial and cytochrome complex assembly which boosts ATP production to meet higher energy demands, while tight regulation of intracellular calcium and other ion homeostasis supports critical signaling and enzyme functions; concurrently, increased expression of genes involved in chaperone-mediated protein folding is observed. Component k = 2 (cyan, spot B) is related to heightened metabolic activity, with primary and organic substance metabolic processes driving fundamental cellular functions. Additionally, the enrichment of molecular functions such as flavin adenine dinucleotide (FAD) binding and adaptor activities underscores the fine-tuning of enzymatic reactions and protein interactions critical for maintaining efficient metabolic pathways and intracellular communication, collectively supporting growth, biosynthesis, and overall cellular homeostasis.

Component k = 3 (blue, spot C) indicates upregulation of translation, peptide biosynthetic, and protein metabolic processes, which suggests a robust production and turnover of proteins, essential for growth and adaptation, while increased amide biosynthetic activity supports the formation of peptide bonds. Concurrently, the response to organonitrogen compounds highlights the plant’s sensitivity to nitrogen-containing molecules, critical for synthesizing amino acids and nucleotides. Moreover, the enrichment of cellular components such as the Set1C/COMPASS and histone methyltransferase complexes points to active chromatin remodeling and epigenetic regulation, ensuring precise control over gene expression. Collectively, these changes reflect a coordinated effort to optimize protein production, maintain cellular organization, and regulate gene activity in response to both internal metabolic demands and external environmental cues. Component k = 4 (green, spots D, E) reflects the pivotal role of membrane-bound and intracellular organelles—integral anatomical entities that compartmentalize and optimize biochemical processes—ensuring that activities such as enzymatic reactions, gene regulation, and signal transduction occur efficiently within defined subcellular locales. Hence, spot segmentation using SOM provides lists of SNPs and genes referring to the genetic components of admixture analysis and, in the next step, enables function mining using gene set enrichment methods.

### Admixture and SOM topology reflect footprints of dissemination of cultivated grapes

**Fig 8:**
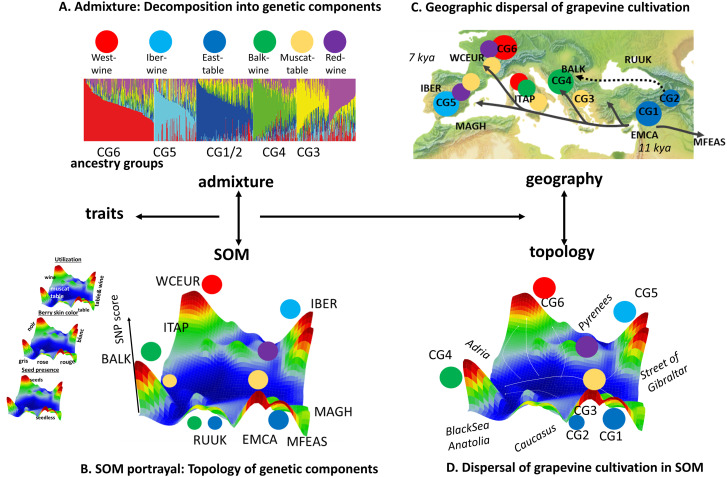
The topology of the grapevine genome. **(A)** Admixture components (colored circles, K = 6) translate into a SOM map expressing topological relations between them **(B)**. **(C)** The components correspond to genetic clusters describing the dissemination paths of cultivated grapes (CG1 – CG6) across the Mediterranean world (arrows) [33]. **(D)** SOM map shows topological similarities with the geographic landscape. Traits and phenotypes are associated with the maxima of the SNP-score. Hence, the topology of the grapevine genome as obtained by SOM-analysis reflects the dissemination of vine in space and time around the ancient Mediterranean world. Basis map of Europe in part C was taken under free license from https://www.shadedreliefarchive.com/Kenneth_Townsend.html.

The history of grape cultivation combines local adaptation with widespread vegetative propagation and movement, with varieties that have achieved broad or worldwide distribution and others that have largely remained confined in narrow geographic areas [38]. The admixture components extracted in our analysis roughly correspond to the six genetic clusters of cultivated grapevines (CG1–CG6), as defined by whole-genome sequencing in relation to their geographic origins and trait characteristics [33] (Fig 8A). Peaks of high SNP scores in the SOM are arranged in a topology-aware manner, indicating that progressively diverging profiles are associated with increasingly distant peak features (Fig 8B). The CG1 and CG2 clusters reflect early domestication footprints originating from Western Asia/Levante (CG1) and the South Caucasus (CG2) approximately 11,000 years ago during the post-glacial warming period. Because of the limited number of accessions from this region in our data set compared with the one used in [2] CG1 and CG2 are not readily decomposed. However, cultivars originating from the northern part of the Caucasus and Black Sea (todays Russia and Ukraine, RUUK) and partly Balkans (inclusive Moldova) carry traces of CG2 with specific spot modules in the SOM. The dissemination of cultivated vine originating from CG1 extended through Anatolia, the Balkans, and Italy (CG3 and CG4), reaching the Iberian Peninsula (CG5) and Western Europe (CG6), where cultivated grapevines arrived approximately 6,000–7,000 years ago (Fig 8C) [33]. Cultivars from the Italian peninsula (ITAP) express a specific spot pattern distinguishable from all the other European populations due to historical events in the area linking South Italian and Greek genotypes highlighting the Greek role as a “bridge” between the Western and Eastern Eurasia [36,40]. The “Red Wine” genetic component (k = 6) admixes cultivars from IBER and WCEU including French and German cultivars possibly related to closer proximities between Portuguese and French vines compared with Spanish ones [38]. The SOM thus provides a “footprinting” approach in gene space that broadly reflects the geographic relatedness among the CG1–6 genetic components. In this way, SOM portrayal complements the decomposition of admixture components by organizing them within a spatially coherent landscape informed by genomic topology.

## Discussion

Linking specific genomic variations to selective traits is a key task for many fields from ecology, plant and animal breeding. The quantitative phenotypic variability found in natural populations is due to a complex underlying genetic interplay of multiple, often unknown, loci with allelic effects affected by environmental conditions. Similarly, a large number of selected traits in breeds of domesticated species occur via the evolution of quantitative, polygenic traits. In those cases, identifying all the genomic variations underlying these traits is highly challenging and motivated the development of a variety of methods.

The major methodical issue we addressed here is the question about the relation between well-proven and frequently used admixture decomposition of genetic components in multi-locus population studies and clustering analysis of the multi-locus genetic features using machine learning based SOM portrayal. Hypothesizing that genetic component Q-values correlate with genetic score values identified in different spots of the SOM we found that SOM portrayal identifies the genetic admixture components as modules of co-mutated SNPs in the population and distributes them in a topology-aware genetic landscape. Topology-awareness hereby means that genetic modules appear as “spot-like” features in a two-dimensional map where they arrange according to their mutual similarities. SOM portrayal thus extends and complements the information content of admixture analysis by providing an easy-to-percept genetic landscape which visualizes the relatedness between the genetic components in terms of a network-like structure. Moreover, portrayal means that these landscapes can be generated for each individual sample as well as for subgroups of them referring to selected traits, properties and geographic regions which enables their association with the genetic components. Finally, the SNP-feature related landscape can be complemented by sample-related topologies thus meeting another important aspect of population analysis.

We here investigated the population genetics of the European grapevine based on whole genome SNP-data published previously [31,32] as a case study. Up to six genetic components were considered in agreement with previous studies [33,35,36,38,40] which considers the genetic diversity of grapevine across the geographic region with sufficient granularity [31]. Namely, the components accumulate into clusters of preferential geographic origin and utilization of the grapes such as East-table and West-wine grapes, wine grapes from Balkans and Iberia as well as more mixed groups referring to Muscat flavor and red Berry skin. Interestingly, these groups reveal correspondence with ancestral and geographic clusters of grapevine derived from whole genome sequencing data of more than 2,000 vine accessions collected worldwide [33]. The topology of the genetic landscape resembles the geographic map of the classical Mediterranean world ranging from the Georgian kingdom of Iberia and the Armenian highlands in the South Caucasus as well as the Levante to the Iberian peninsula, Maghreb and Western Europe. The genetic map shows distinct “mountain”-tips of high SNP-score for the major genetic admixture components which typically collect a few hundred specific SNPs each. The genetic topology reflects the fact that grapevine domestication and subsequent dissemination appeared dynamic in space and time, defining a continuum from wild to cultivated populations, via incipient domesticated populations [38].

With increasing k > 4 the distribution of SNPs becomes progressively diffuse and covers areas of the major components k = 1–4: For example, the Muscat table group overlaps with regions of East table and Iber wine while the Red wine group overlaps with the West wine and Iber wine areas in agreement with [33]. These results show that the vine genomes only partly can be described by disjunct, clear-cut genetic clusters but instead revealed mutual admixtures and more continuous distribution characteristics. The CV-error plot suggests maximum K greater 6, which however seems to overfit our data: (i) a parallel study on 2000 vine accessions also cut the maximum K despite the respective CV didn’t converge thus enabling the straightforward interpretation of the admixture components [33], (ii) studies on the population structure on other organisms that use larger K > 9 referring to the minimum of CV often show volatile and hardly to interpret admixture results (see, e.g., [41]), (iii) SOM analysis clearly indicates convergence of the reasonable components for K > 4 based on the silhouette score, the observed spot patterns as well as the variance structure of the components (Figs 5 - 7). Hence, a “less is more” approach to selecting the maximum K seems advisable, particularly when the distributions of Q-amplitudes display a broad continuum of values. Parallel SOM analysis therefore offers an additional assessment of reasonable and interpretable K values. In this context, the SOM provides another evaluation strategy by using the silhouette score as well as the visual inspection of incremental spot patterns as K increases (Figs 3, 6, and 7). Moreover, SOM spot analysis enables the direct extraction of SNPs, genes, and the associated functions of genetic components. Consequently, SOMmelier not only visualizes the topology of the genetic landscape but also complements admixture analysis by providing detailed information about the variability of additional genetic components and their functional roles.

A limitation of SOMmelier is that it is not fully automated and requires subjective input and domain expertise, for example in defining sample groups. In this context, admixture analysis provides a synergistic approach by enabling genetic stratification prior to SOM analysis. Interpretation of SOM results likewise requires domain knowledge; however, this is supported by the extensive auxiliary information generated by the software, which facilitates interpretation of SOM visualizations (see above and [22,23]).

In this study, we used a large grapevine dataset as a working example, raising the question of the method’s generalizability to other genetic and omics data beyond the vine genome. Previously, we successfully applied SOM analysis to human data from the 1000 Genomes Project [29] and to approximately 65,000 SARS-CoV-2 genomes [28], demonstrating its applicability across diverse genetic datasets, particularly for generating genomic landscapes and evolutionary relations. More broadly, SOM-based portrayals have been applied to a wide range of omics data, including copy number variation, transcriptomic, methylome, histone modification, and proteomic datasets as well as data from humans, animals, cell models, and plants (see references provided in the S1 Text). These applications further underscore the versatility of the approach, while also high-lighting the need for domain-specific expertise and tailored downstream analyses in each case.

## Conclusions

SOMmelier is a powerful method that complements and extends admixture analysis to study genetic variation. It can be used to establish the multidimensional relatedness between admixture proportions to embed population differentiation due to processes such as genetic drift, migration, and mutation. The grapevine genomes illustrate the adaptive divergence of ancestral populations at their initial locations, with putative differential introgression. The use of admixture components, that integrate the effects of demography and of natural selection, can thereby explain phenotypic variation in terms of a topological map. We see SOMmelier as a method that extends genetic admixture analyses in a wider application range beyond the grapevine. Another interesting and challenging field is the more detailed analysis of grapevine accessions from the Caucasus under consideration of wild vine (Vitis sylvestris) accessions to establish a genetic map of one of cultivation centers of cultivated vines.

## Materials and methods

### SNP microarray data of worldwide vitis vinifera accessions

**Fig 9:**
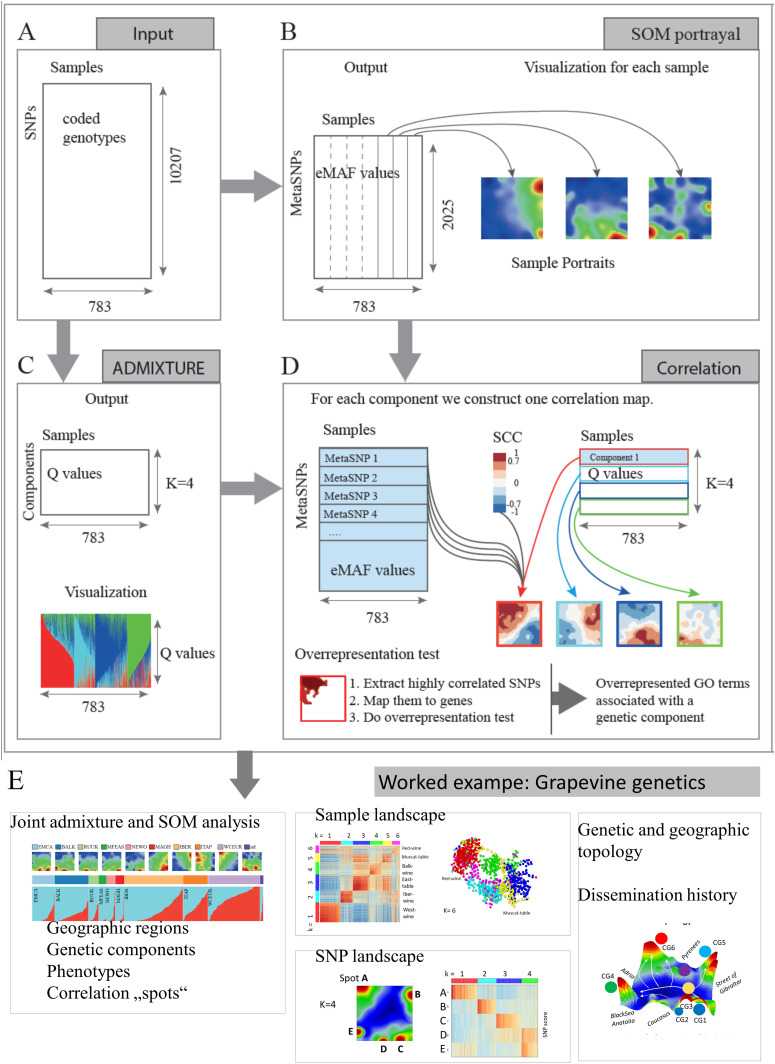
Workflow of combined Admixture and SOM analysis for interpretation of genetic components. **A)** Input data is a G (SNPs) x S (samples) genotype matrix (10,207 x 783 in case of worldwide data) **B)** SOM training delivers a matrix of reduced dimensionality, where each meta-SNP is a cluster of single SNPs. Its value can be interpreted as the estimated mean eMAF (excess minor allele frequency) value averaged over the single SNPs in each meta-SNP. Each column (sample) is visualized as a SOM portrait by arranging the meta-SNP values in a square grid and coloring them according to their value in max-min scale from red (max) to blue (min). **C)** ADMIXTURE conducts population structure analysis and provides percentages (Q-values) of the genetic compounds. The analysis is done for a range of K, here for K = 4. **D)** Each Q-value profile is correlated with all meta-SNP profiles using Spearman’s Correlation Coefficient, ⍴(rho), providing a “correlation portrait” by coloring the correlation coefficients from red (max, i.e., positive) to blue (min, i.e., negative). Gene lists were extracted from areas of high values and used for GO function mining. **E)** Worked example presented in this publication: Combined admixture and SOM analysis is used to extract genetic components (see **D)**. The SOM splits into a sample and SNP landscape which reflect the topology of the vine genome under different aspects.

We analyzed SNP microarray and phenotype data from worldwide accessions obtained from a previous study [31]. This dataset was generated using the GrapeReSeq 18K Vitis genotyping microarray, which probed 10,207 SNPs across 783 accessions collected globally (Fig 9A). For downstream analysis, the data matrix was transformed into a PLINK binary biallelic genotype table format and subsequently analyzed using ADMIXTURE. The genotype matrix was also converted into a numeric format to facilitate Self-Organizing Map (SOM) representation, as described in a previous study [32]: Genotypes were coded as 0, 1, and 2, corresponding to homozygous reference, heterozygous, and homozygous alternative alleles, respectively. Phenotypic data included traits such as fruit utilization, flower sex (International Organization of Vine and Wine identifier: OIV-151), berry skin color (OIV-225), seed presence or absence (OIV-241), muscat flavor (OIV-236), phenological characteristics (OIV-301 to OIV-304), fertility (OIV-155), bunch and berry weights (OIV-502 and OIV-503), susceptibility of bunches to botrytis (OIV-459), and must acidity (OIV-506).

### ADMIXTURE analysis

To determine the ancestral composition of the worldwide grapevine accessions, we employed the ADMIXTURE tool ([42], version 1.3.0, accessed September 15^th^ 2024) with independent runs for different numbers of genetic components, K = 2…6 and default parameter settings. The primary output of ADMIXTURE is the Q-matrix (Fig 9B), an S × K table where S represents the number of samples and K denotes the assumed number of ancestral populations. This matrix contains Q-values, which correspond to the estimated composition of ancestral components for each sample. These values are visualized in the ADMIXTURE stacked barplots, providing the Q-composition of each cultivar. The cross-validation (CV) error calculation feature was taken from the ADMIXTURE runs to assess model accuracy and identify the most suitable value of K.

### Self organizing maps for genome portrayal of grapevine: SOMmelier

To perform dimensionality reduction and clustering, we used the Self-Organizing Maps (SOM) machine learning algorithm, implemented in the OposSOM R package [22] (accessed September 15^th^, 2025, Fig 9C). The SOM algorithm was originally invented by Toivo Kohonen as a highly effective unsupervised clustering method based on (iterative) machine learning of the intrinsic covariance structure of complex data [43]. Twenty years later Ingber and colleagues [44] applied SOM to inspect highly resolved gene expression landscapes as two-dimensional images. Another ten years later we further developed this visualization method into a comprehensive analysis pipeline for omics data [23,45] supported by the oposSOM software package [22] and an interactive web-browser [46]. oposSOM has been successfully applied by us in more than 40 own publications and by external users in more than 20 applications until 2020 (see supplementary material in [46]), numbers which have been roughly doubled since then, mainly for applications of bulk and single cell transcriptomic data in the health and cancer context.

Application to genetic SNP data of grapevine required modifications to the preprocessing metrics as well as to the downstream knowledge-mining functionalities, which are collectively referred to here as SOMmelier**,** a derivative of oposSOM (see also S1 Text)**.** For SOM training the SNP values were preprocessed as follows: First they were coded with the integers 0, 1 and 2 for the homozygous major, heterozygous and homozygous minor allele, respectively. Then theses values were centralized by subtracting the mean value from each individual SNP across all samples, which will be referred as a SNP-score. This centralization step provides the SNP-score defined as excess minor allele frequency (eMAF; see [32] and S1 Text) used as input data for SOM training. Ternary integer labeling using “0–1–2” to represent “major–heterozygous–minor” allelic SNP states, followed by centralization of each SNP across all samples, balances the SNP scores around zero. This transformation reflects the deviation of the minor allele frequency of a given SNP in a particular sample relative to its mean frequency across the entire dataset (see S1 Text). Positive values indicate SNPs with a higher minor allele frequency than the population average and negative values indicate SNPs with a smaller minor allele frequency than the population average. Hence, the SNP-score thus defines the excess minor allele frequency (eMAF) as the deviation of MAF relative to its population average. The use of such centralized measures has previously been applied to transcriptomic, methylome, and copy-number data to generate molecular portraits with high sensitivity [25].

The SNP-score values constitute a numeric matrix of size N = G x S with a number of rows which corresponds to the number of SNPs, G, and a number of columns, which corresponds to the number of samples, S. Next, the data matrix was processed by SOM machine learning in its SOM-portrayal version [22,32] which reduces the size of the data to P = Mx S with M denoting the number of metaSNPs, M < G, where each meta-SNP represents a micro-cluster of co-mutated SNPs across the dataset. The SNP-scores of all metaSNP of each sample were visualized as square images called SNP-portraits using a red- green-blue color scale for meta-SNPs with scores referring to minor, heterozygous and major homozygous alleles, respectively. In the portraits, similar meta-SNP profiles cluster together, forming spot-like red (and blue) areas referring to modules of co-mutated SNPs in the population studied due to the self-organizing properties of the SOM algorithm [23]. SNPs accumulating in these areas were extracted by “overexpression” criteria as implemented in oposSOM/SOMmelier and used for functional interpretation by means of gene set enrichment analysis [22]. We analyzed S = 783 vine accessions considering G = 10,207 SNPs per sample to train a SOM of size M = 45x 45 = 2,025 metaSNPs.

### Correlation maps between ADMIXTURE and SOM

To identify the SNPs associated with the admixture components, we calculated the correlation between the Q-value profiles of each admixture component (k = 1, …K) and each of the M meta-SNP profiles, thus providing Kx M Spearman’s correlation coefficients (SCC), where correlation maps of size M were generated for each genetic component, matching the SOM portraits (Fig 9D). From the correlation maps, we identified meta-SNPs with high SCC values ≥0.7, corresponding to the red regions in the maps. These highly correlated meta-SNPs were extracted and subsequently used for functional annotation and enrichment analysis. Variation of the selection criterion will either decrease (e.g., for SCC > 0.8) or increase (e.g., for SCC > 0.5) the number of extracted SNPs. Our threshold was chosen to ensure separation of detected areas in the SOM between the admixture components and to enable function mining which usually requires from a few dozen to a few hundred SNPs/genes.

### Functional analysis

The extracted highly correlated meta-SNPs from the maps of each admixture component were converted back to SNP thus providing lists of SNPs associated with the respective genetic component. Next, we used ANNOVAR [47] (accessed September 25^th^ 2024)to annotate the SNPs and selected the SNPs which mapped to genes. As a result, we obtained lists of genes associated with each component. Gene set overrepresentation analysis was conducted for these gene lists using Fisher’s exact test and setting a significance level of 0.05 for adjusted p-values. Gene ontology (GO) enrichment analysis was conducted for these gene lists using the Overrepresentation Test of PANTHER [48] using default settings (accessed in January 10th 2025), followed by the Semantic similarity reduction feature of REVIGO [49] (accessed in January 10^th^ 2025; used with default settings).

## Supporting information

S1 Fig SOM portrayal of the genomes of grapevine accessions. This study presents the Self-Organizing Map (SOM) representation of 783 grapevine accessions, categorized according to the K = 6 admixture components. Individual portraits of all accessions are displayed, alongside larger maps representing the mean portrait for each group. The mean portraits are computed by averaging all individual portraits within a group and are presented in two distinct color scales: (i) a standard color scale, which highlights the regions with maximum and minimum SNP-scores in red and blue, respectively, and (ii) a “waterline” scale, where negative and positive SNP-scores are depicted in blue and red, respectively. It is important to note that the SNP-score is centralized, meaning that positive and negative values indicate deviations from the mean SNP-score of each SNP across all accessions. Additionally, a spot co-expression map is provided, connecting co-expressed spots within individual portraits through lines, thereby visualizing the co-mutation networks within each group. Together, the three group-related portraits provide complementary visualizations of the genetic components’ topology. The standard mean portraits emphasize characteristic spot patterns corresponding to high and low SNP-scores, while the waterline representation more effectively highlights regions of SNP-scores. slightly above (red) and below (blue) zero. Meanwhile, the spot co-expression map segments portraits into distinct spot patterns and illustrates overlapping co-mutation networks, demonstrating that SNPs with high scores may be shared a cross multiple groups.
(TIF)

S2 Fig Phenotype enrichment of the genetic admixture components. The curves show the percentage of grapevine accessions of the respective phenotypes in each of the six genetic components, averaged over a sliding window of 20 accessions moving from left to right. (see also Fig 4). The averaging better expresses systematic changes of phenotype composition as a function of the genetic components. Geographic region: The group “nicknames” were given according to the dominating geographic contribution, e.g., WCEUR and IBER dominate across the red and light-blue components, respectively, while MFEAS and EMCA both contribute to the East-table accessions. Muscat-table distributes over a wider geographic range. Italian vines (ITAB) increase in their fraction in more diverse regions of West-wine and BALK-wine, thus revealing similarities with neighboring regions. Utilization: Table utilization is associated with the blue and yellow components. Berry skin color: The magenta component combines predominantly red wines from WCEUR and IBER. Seed presence: Seedless accessions are associated with table utilization.
(TIF)

S3 Fig Sample similarity plots. (A) Phylogenetic tree presentation for K = 4 (left part) and K = 6 (right). The four major components distribute virtually along distinct branches while the additional fifth (yellow, Muscat table) and sixth (magenta, Red wine) components either stick to the blue branch (yellow to blue) or form separate branches (magenta). (B) Independent com-ponent analysis distributes the four major components virtually along separate axes as schematically sketched in the insertion, which indicates their partial independence.
(TIF)

S4 Fig Quantifying the topology of the SOM landscape of grapevine. (A) The density distribution of the SNP-scores stratified to K = 6. The three peaks refer to major homozygous, heterozygous and minor homozygous alleles (left to right). Note that the SNP-score initially codes them with 0, 1 and 2, respectively, and subsequently centralizes them for each SNP by subtracting the mean value calculated over all samples of the study. Con-sequently, the zero-value of the score refers to the mean value of the SNP-code, the peak at negative values to major allelic SNPs, and the rightmost peak to exclusively minor allelic SNPs across all accessions. The color code of the SOM portraits changes smoothly from the left to the right by assigning typically major allelic, heterozygous and minor allelic SNPs to blue, green and red, respectively. “Spots” were identified using a threshold of SNP-score applied to each meta-SNP and labelled with capital letters. See also the S1 Text section for the definition and calculation of the SNP-score. (B) The personalized spot summary maps plot all spot areas detected in the individual portraits of the accessions. The spot-segmentation map shows all spots as colored areas. C) The three supporting maps visualize the variance per metagene (variance map), the Euclidean distance between neigh-boring metagenes (D-map) and population (number of SNPs) per metagene. Areas of high variance (maroon) mostly agree with “crater-like” structures which consist of a red ring (expanded distances) around a white dot in the middle (reduced distance). Note that SOM training adjusts the distances between the metagenes to better resolve high-variant regions. The blue areas in both maps refer to SNPs with virtually invariant SNP-scores. The population map indicates that highly pop-ulated metaSNPs arrange near the edges and corners of the map. Empty metaSNPs form borderlines between regions of different co-variance structure, particularly separating invariant regions from variant ones as red lines). (D) Spot co-expression in the individual portraits of each group are visualized by lines connecting the respective spots. The co-expression networks predominantly include spots in the regions overexpressed in the respective mean portraits (compare with S1 Fig). In addition, the networks better resolve the heterogeneity of spot co-expression between the groups, especially for k > 3. The heatmaps show the SNP-expression of the spots for k = 4 (left) and k = 6 (right), which become increasingly unspecific regarding the different groups. The letters serve as a visual guide for different spot groups.
(TIF)

S1 Text Supplementary Methods. (DOCX)

S1 Table Highly correlated SNPs and genes for K = 4. (XLSX)

S2 Table Highly correlated SNPs and genes for K = 6. (XLSX)

S3 Table SNPs in spots for K = 4. (XLSX)

S4 Table SNPs in spots for K = 6. (XLSX)

S5 Table Overrepresentation analysis for K = 4. (XLSX)

S6 Table Overrepresentation analysis for K = 6. (XLSX)

## References

[1] Wang J. Fast and accurate population admixture inference from genotype data from a few microsatellites to millions of SNPs. Heredity (Edinb). 2022;129(2):79–92. doi: 10.1038/s41437-022-00535-z 35508539 PMC9338324

[2] Pritchard JK, Stephens M, Donnelly P. Inference of population structure using multilocus genotype data. Genetics. 2000;155(2):945–59. doi: 10.1093/genetics/155.2.945 10835412 PMC1461096

[3] Alexander DH, Novembre J, Lange K. Fast model-based estimation of ancestry in unrelated individuals. Genome Res. 2009;19(9):1655–64. doi: 10.1101/gr.094052.109 19648217 PMC2752134

[4] Lawson DJ, van Dorp L, Falush D. A tutorial on how not to over-interpret STRUCTURE and ADMIXTURE bar plots. Nat Commun. 2018;9(1):3258. doi: 10.1038/s41467-018-05257-7 30108219 PMC6092366

[5] Kaeuffer R, Réale D, Coltman DW, Pontier D. Detecting population structure using STRUCTURE software: effect of background linkage disequilibrium. Heredity (Edinb). 2007;99(4):374–80. doi: 10.1038/sj.hdy.6801010 17622269

[6] Novembre J. Pritchard, Stephens, and Donnelly on Population Structure. Genetics. 2016;204(2):391–3. 27729489 10.1534/genetics.116.195164 PMC5068833

[7] Padakanti S, Tiong K-L, Chen Y-B, Yeang C-H. Genotypes of informative loci from 1000 Genomes data allude evolution and mixing of human populations. Sci Rep. 2021;11(1):17741. doi: 10.1038/s41598-021-97129-2 34493766 PMC8423758

[8] Patterson N, Price AL, Reich D. Population structure and eigenanalysis. PLoS Genet. 2006;2(12):e190. doi: 10.1371/journal.pgen.0020190 17194218 PMC1713260

[9] Price AL, Patterson NJ, Plenge RM, Weinblatt ME, Shadick NA, Reich D. Principal components analysis corrects for stratification in genome-wide association studies. Nat Genet. 2006;38(8):904–9. doi: 10.1038/ng1847 16862161

[10] Jombart T, Devillard S, Balloux F. Discriminant analysis of principal components: a new method for the analysis of genetically structured populations. BMC Genet. 2010;11:94. doi: 10.1186/1471-2156-11-94 20950446 PMC2973851

[11] Diaz-Papkovich A, Anderson-Trocmé L, Gravel S. A review of UMAP in population genetics. J Hum Genet. 2021;66(1):85–91. doi: 10.1038/s10038-020-00851-4 33057159 PMC7728596

[12] Novembre J, Stephens M. Interpreting principal component analyses of spatial population genetic variation. Nat Genet. 2008;40(5):646–9. doi: 10.1038/ng.139 18425127 PMC3989108

[13] McVean G. A genealogical interpretation of principal components analysis. PLoS Genet. 2009;5(10):e1000686. doi: 10.1371/journal.pgen.1000686 19834557 PMC2757795

[14] Lazaridis I, Patterson N, Anthony D, Vyazov L, Fournier R, Ringbauer H, et al. The genetic origin of the Indo-Europeans. Nature. 2025 639(8053):132–42. 39910300 10.1038/s41586-024-08531-5 PMC11922553

[15] Haak W, Lazaridis I, Patterson N, Rohland N, Mallick S, Llamas B, et al. Massive migration from the steppe was a source for Indo-European languages in Europe. Nature. 2015;522(7555):207–11. doi: 10.1038/nature14317 25731166 PMC5048219

[16] Librado P, Khan N, Fages A, Kusliy MA, Suchan T, Tonasso-Calvière L, et al. The origins and spread of domestic horses from the Western Eurasian steppes. Nature. 2021;598(7882):634–40. doi: 10.1038/s41586-021-04018-9 34671162 PMC8550961

[17] Wang GD, Zhai W, Yang HC, Fan RX, Cao X, Zhong L, et al. The genomics of selection in dogs and the parallel evolution between dogs and humans. Nature Communications. 2013;4(1):1860. 10.1038/ncomms2814 23673645

[18] Nilson SM, Gandolfi B, Grahn RA, Kurushima JD, Lipinski MJ, Randi E, et al. Genetics of randomly bred cats support the cradle of cat domestication being in the Near East. Heredity (Edinb). 2022;129(6):346–55. doi: 10.1038/s41437-022-00568-4 36319737 PMC9708682

[19] Razifard H, Ramos A, Della Valle AL, Bodary C, Goetz E, Manser EJ, et al. Genomic evidence for complex domestication history of the cultivated tomato in Latin America. Molecular Biology and Evolution. 2020;37(4):1118–32. doi: 10.1093/molbev/msz303 31912142 PMC7086179

[20] He W, Chen C, Xiang K, Wang J, Zheng P, Tembrock LR, et al. The history and diversity of rice domestication as resolved from 1464 complete plastid genomes. Front Plant Sci. 2021;12. 10.3389/fpls.2021.781793 PMC8637288 34868182

[21] Luo X, Zhou H, Cao D, Yan F, Chen P, Wang J, et al. Domestication and selection footprints in Persian walnuts (Juglans regia). PLoS Genet. 2022;18(12):e1010513. doi: 10.1371/journal.pgen.1010513 36477175 PMC9728896

[22] Löffler-Wirth H, Kalcher M, Binder H. oposSOM: R-package for high-dimensional portraying of genome-wide expression landscapes on bioconductor. Bioinformatics. 2015;31(19):3225–7. doi: 10.1093/bioinformatics/btv342 26063839

[23] Wirth H, Löffler M, von Bergen M, Binder H. Expression cartography of human tissues using self organizing maps. BMC Bioinformatics. 2011;12:306. doi: 10.1186/1471-2105-12-306 21794127 PMC3161046

[24] Loeffler-Wirth H, Kreuz M, Hopp L, Arakelyan A, Haake A, Cogliatti SB, et al. A modular transcriptome map of mature B cell lymphomas. Genome Med. 2019;11(1):27. doi: 10.1186/s13073-019-0637-7 31039827 PMC6492344

[25] Binder H, Schmidt M, Hopp L, Davitavyan S, Arakelyan A, Loeffler-Wirth H. Integrated Multi-Omics Maps of Lower-Grade Gliomas. Cancers (Basel). 2022;14(11):2797. doi: 10.3390/cancers14112797 35681780 PMC9179546

[26] Hopp L, Löffler-Wirth H, Galle J, Binder H. Combined SOM-portrayal of gene expression and DNA methylation landscapes disentangles modes of epigenetic regulation in glioblastoma. Epigenomics. 2018;10(6):745–64. doi: 10.2217/epi-2017-0140 29888966

[27] Hopp L, Loeffler-Wirth H, Nersisyan L, Arakelyan A, Binder H. Footprints of Sepsis Framed Within Community Acquired Pneumonia in the Blood Transcriptome. Front Immunol. 2018;9(1620). 10.3389/fimmu.2018.01620 PMC6056630 30065722

[28] Schmidt M, Arshad M, Bernhart SH, Hakobyan S, Arakelyan A, Loeffler-Wirth H, et al. The Evolving Faces of the SARS-CoV-2 Genome. Viruses. 2021;13(9):1764. 34578345 10.3390/v13091764 PMC8472651

[29] Nikoghosyan M, Hakobyan S, Hovhannisyan A, Loeffler-Wirth H, Binder H, Arakelyan A. Population levels assessment of the distribution of disease-associated variants with emphasis on Armenians – a machine learning approach. Front Genet. 2019;10(394). 10.3389/fgene.2019.00394 PMC6498285 31105750

[30] Binder H, Wirth H. Analysis of large-scale OMIC data using Self Organizing Maps. In: Khosrow-Pour M, editor. Encyclopedia of Information Science and Technology Third Edition. IGI Global; 2014. p. 1642–54.

[31] Laucou V, Launay A, Bacilieri R, Lacombe T, Adam-Blondon A-F, Bérard A, et al. Extended diversity analysis of cultivated grapevine Vitis vinifera with 10K genome-wide SNPs. PLoS One. 2018;13(2):e0192540. doi: 10.1371/journal.pone.0192540 29420602 PMC5805323

[32] Nikoghosyan M, Schmidt M, Margaryan K, Loeffler-Wirth H, Arakelyan A, Binder H. SOMmelier—Intuitive Visualization of the Topology of Grapevine Genome Landscapes Using Artificial Neural Networks. Genes (Basel). 2020;11(7):817. doi: 10.3390/genes11070817 32709105 PMC7397337

[33] Dong Y, Duan S, Xia Q, Liang Z, Dong X, Margaryan K, et al. Dual domestications and origin of traits in grapevine evolution. Science. 2023;379(6635):892–901. doi: 10.1126/science.add8655 36862793

[34] Magaryan K, Nikogհosyan M, Baloyan A, Gasoyan H, Hovhannisyan E, Galstyan L, et al. Machine learned-based visualization of the diversity of grapevine genomes worldwide and in Armenia using SOMmelier. BIO Web Conf. 2023;68:01009. doi: 10.1051/bioconf/20236801009

[35] Ramos-Madrigal J, Runge AKW, Bouby L, Lacombe T, Samaniego Castruita JA, Adam-Blondon A-F, et al. Palaeogenomic insights into the origins of French grapevine diversity. Nat Plants. 2019;5(6):595–603. doi: 10.1038/s41477-019-0437-5 31182840

[36] Mercati F, De Lorenzis G, Mauceri A, Zerbo M, Brancadoro L, D’Onofrio C, et al. Integrated Bayesian Approaches Shed Light on the Dissemination Routes of the Eurasian Grapevine Germplasm. Front Plant Sci. 2021;12. 10.3389/fpls.2021.692661 PMC8381769 34434204

[37] Bouby L, Wales N, Jalabadze M, Rusishvili N, Bonhomme V, Ramos‑Madrigal J, et al. Correction to: Tracking the history of grapevine cultivation in Georgia by combining geometric morphometrics and ancient DNA. Veget Hist Archaeobot. 2022;31(3):329–329. doi: 10.1007/s00334-022-00872-3

[38] Magris G, Jurman I, Fornasiero A, Paparelli E, Schwope R, Marroni F, et al. The genomes of 204 Vitis vinifera accessions reveal the origin of European wine grapes. Nat Commun. 2021;12(1):7240. doi: 10.1038/s41467-021-27487-y 34934047 PMC8692429

[39] Sargolzaei M, Rustioni L, Cola G, Ricciardi V, Bianco PA, Maghradze D, et al. Georgian Grapevine Cultivars: Ancient Biodiversity for Future Viticulture. Front Plant Sci. 2021;12:630122. doi: 10.3389/fpls.2021.630122 33613611 PMC7892605

[40] De Lorenzis G, Mercati F, Bergamini C, Cardone MF, Lupini A, Mauceri A, et al. SNP genotyping elucidates the genetic diversity of Magna Graecia grapevine germplasm and its historical origin and dissemination. BMC Plant Biol. 2019;19(1):7. doi: 10.1186/s12870-018-1576-y 30612542 PMC6322315

[41] Stronen AV, Pertoldi C, Iacolina L, Kadarmideen HN, Kristensen TN. Genomic analyses suggest adaptive differentiation of northern European native cattle breeds. Evol Appl. 2019;12(6):1096–113. doi: 10.1111/eva.12783 31293626 PMC6597895

[42] Alexander DH, Lange K. Enhancements to the ADMIXTURE algorithm for individual ancestry estimation. BMC Bioinformatics. 2011;12:246. doi: 10.1186/1471-2105-12-246 21682921 PMC3146885

[43] Kohonen T. Self-organized formation of topologically correct feature maps. Biol Cybern. 1982;43(1):59–69. doi: 10.1007/bf00337288

[44] Eichler GS, Huang S, Ingber DE. Gene Expression Dynamics Inspector (GEDI): for integrative analysis of expression profiles. Bioinformatics. 2003;19(17):2321–2. doi: 10.1093/bioinformatics/btg307 14630665

[45] Wirth H, von Bergen M, Binder H. Mining SOM expression portraits: feature selection and integrating concepts of molecular function. BioData Min. 2012;5(1):18. doi: 10.1186/1756-0381-5-18 23043905 PMC3599960

[46] Loeffler-Wirth H, Reikowski J, Hakobyan S, Wagner J, Binder H. oposSOM-Browser: an interactive tool to explore omics data landscapes in health science. BMC Bioinformatics. 2020;21(1):465. doi: 10.1186/s12859-020-03806-w 33076824 PMC7574456

[47] Wang K, Li M, Hakonarson H. ANNOVAR: functional annotation of genetic variants from high-throughput sequencing data. Nucleic Acids Res. 2010;38(16):e164. doi: 10.1093/nar/gkq603 20601685 PMC2938201

[48] Thomas PD, Ebert D, Muruganujan A, Mushayahama T, Albou L-P, Mi H. PANTHER: Making genome-scale phylogenetics accessible to all. Protein Sci. 2022;31(1):8–22. doi: 10.1002/pro.4218 34717010 PMC8740835

[49] Supek F, Bošnjak M, Škunca N, Šmuc T. REVIGO summarizes and visualizes long lists of gene ontology terms. PLoS One. 2011;6(7):e21800. doi: 10.1371/journal.pone.0021800 21789182 PMC3138752

