# Supplementary figures and images for: A topological map of the genetic components of grapevine—Admixture meets SOMmelier machine learning
Source: PLoS Comput Biol. 2026 Feb 20;22(2):e1013882. doi: 10.1371/journal.pcbi.1013882 (PMC12948125; doi:10.1371/journal.pcbi.1013882)

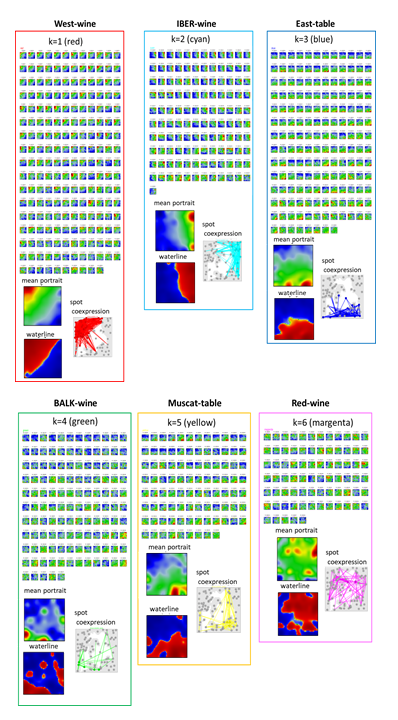

Supplement: S1 Fig — This study presents the Self-Organizing Map (SOM) representation of 783 grapevine accessions, categorized according to the K = 6 admixture components. Individual portraits of all accessions are displayed, alongside larger maps representing the mean portrait for each group. The mean portraits are computed by averaging all individual portraits within a group and are presented in two distinct color scales: (i) a standard color scale, which highlights the regions with maximum and minimum SNP-scores in red and blue, respectively, and (ii) a “waterline” scale, where negative and positive SNP-scores are depicted in blue and red, respectively. It is important to note that the SNP-score is centralized, meaning that positive and negative values indicate deviations from the mean SNP-score of each SNP across all accessions. Additionally, a spot co-expression map is provided, connecting co-expressed spots within individual portraits through lines, thereby visualizing the co-mutation networks within each group. Together, the three group-related portraits provide complementary visualizations of the genetic components’ topology. The standard mean portraits emphasize characteristic spot patterns corresponding to high and low SNP-scores, while the waterline representation more effectively highlights regions of SNP-scores. slightly above (red) and below (blue) zero. Meanwhile, the spot co-expression map segments portraits into distinct spot patterns and illustrates overlapping co-mutation networks, demonstrating that SNPs with high scores may be shared a cross multiple groups. (TIF) [file pcbi.1013882.s001.tif]

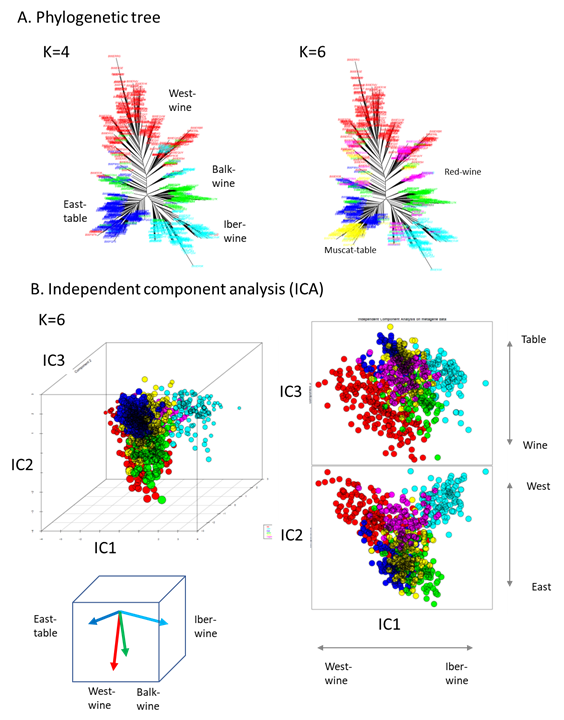

Supplement: S2 Fig — The curves show the percentage of grapevine accessions of the respective phenotypes in each of the six genetic components, averaged over a sliding window of 20 accessions moving from left to right. (see also Fig 4). The averaging better expresses systematic changes of phenotype composition as a function of the genetic components. Geographic region: The group “nicknames” were given according to the dominating geographic contribution, e.g., WCEUR and IBER dominate across the red and light-blue components, respectively, while MFEAS and EMCA both contribute to the East-table accessions. Muscat-table distributes over a wider geographic range. Italian vines (ITAB) increase in their fraction in more diverse regions of West-wine and BALK-wine, thus revealing similarities with neighboring regions. Utilization: Table utilization is associated with the blue and yellow components. Berry skin color: The magenta component combines predominantly red wines from WCEUR and IBER. Seed presence: Seedless accessions are associated with table utilization. (TIF) [file pcbi.1013882.s002.tif]

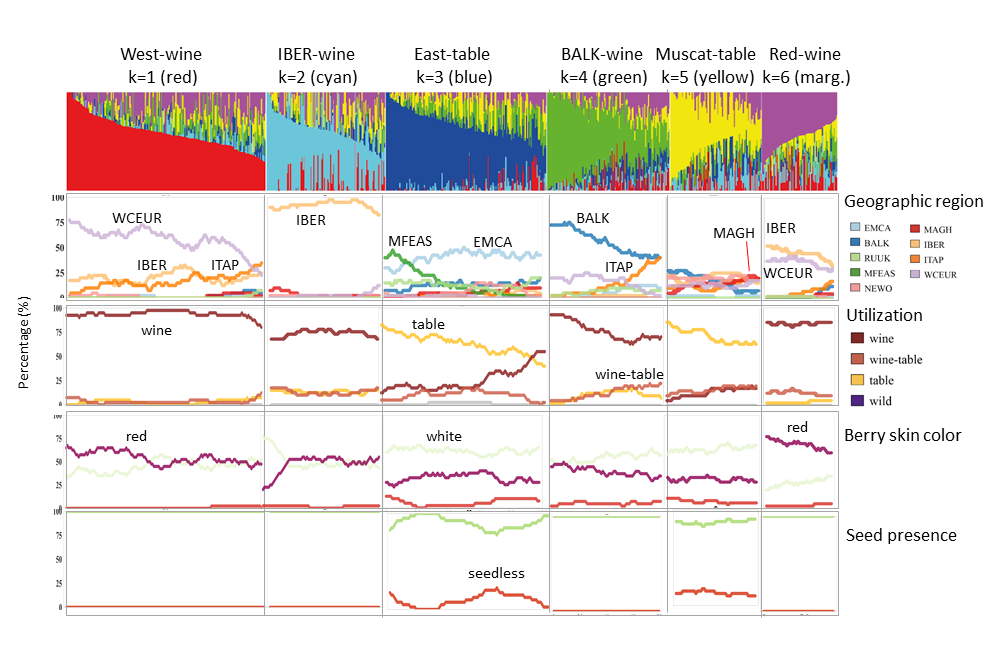

Supplement: S3 Fig — (A) Phylogenetic tree presentation for K = 4 (left part) and K = 6 (right). The four major components distribute virtually along distinct branches while the additional fifth (yellow, Muscat table) and sixth (magenta, Red wine) components either stick to the blue branch (yellow to blue) or form separate branches (magenta). (B) Independent com-ponent analysis distributes the four major components virtually along separate axes as schematically sketched in the insertion, which indicates their partial independence. (TIF) [file pcbi.1013882.s003.tif]

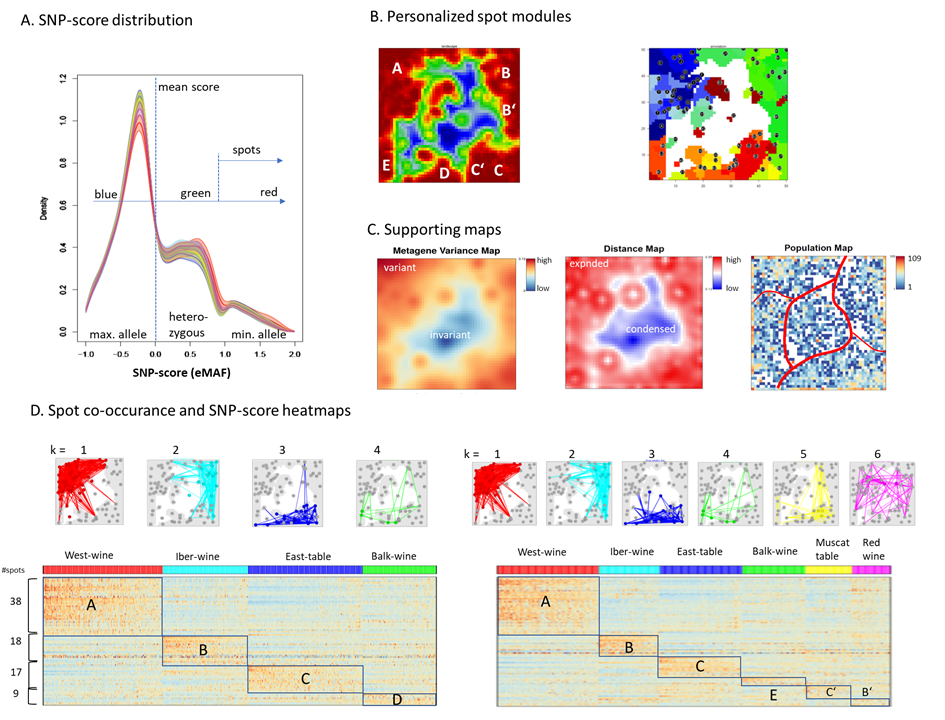

Supplement: S4 Fig — (A) The density distribution of the SNP-scores stratified to K = 6. The three peaks refer to major homozygous, heterozygous and minor homozygous alleles (left to right). Note that the SNP-score initially codes them with 0, 1 and 2, respectively, and subsequently centralizes them for each SNP by subtracting the mean value calculated over all samples of the study. Con-sequently, the zero-value of the score refers to the mean value of the SNP-code, the peak at negative values to major allelic SNPs, and the rightmost peak to exclusively minor allelic SNPs across all accessions. The color code of the SOM portraits changes smoothly from the left to the right by assigning typically major allelic, heterozygous and minor allelic SNPs to blue, green and red, respectively. “Spots” were identified using a threshold of SNP-score applied to each meta-SNP and labelled with capital letters. See also the S1 Text section for the definition and calculation of the SNP-score. (B) The personalized spot summary maps plot all spot areas detected in the individual portraits of the accessions. The spot-segmentation map shows all spots as colored areas. C) The three supporting maps visualize the variance per metagene (variance map), the Euclidean distance between neigh-boring metagenes (D-map) and population (number of SNPs) per metagene. Areas of high variance (maroon) mostly agree with “crater-like” structures which consist of a red ring (expanded distances) around a white dot in the middle (reduced distance). Note that SOM training adjusts the distances between the metagenes to better resolve high-variant regions. The blue areas in both maps refer to SNPs with virtually invariant SNP-scores. The population map indicates that highly pop-ulated metaSNPs arrange near the edges and corners of the map. Empty metaSNPs form borderlines between regions of different co-variance structure, particularly separating invariant regions from variant ones as red lines). (D) Spot co-expression in the indivi [file pcbi.1013882.s004.tif]
