# Supplementary material for: A topological map of the genetic components of grapevine—Admixture meets SOMmelier machine learning
Source: PLoS Comput Biol. 2026 Feb 20;22(2):e1013882. doi: 10.1371/journal.pcbi.1013882 (PMC12948125; doi:10.1371/journal.pcbi.1013882)
Supplement: S1 Text — (DOCX) [file pcbi.1013882.s005.docx]

**Supplementary Methods**

***SOMmelier: Conceptual overview***

Our analysis concept is based on: (i) Transformation of SNP data into excess minor allele frequencies (eMAF) which increases sensitivity for subtle differences between the genomes of the vine accessions; (ii) Dimension reduction of the more than G= 10,207 SNPs into a matrix of M= 2,025 ‘meta-SNPs’ using unsupervised clustering and their visualization in terms of individual accession portraits using SOM machine learning. (iii) Further reduction of dimension by segmentation of the SNP-portraits into about one-to-two dozen so-called spot clusters of correlated SNPs which serve as fingerprint features of the vine genomes; (iv) diversity analysis of accessions using different approaches (principal component analysis, correlation networks, minimum spanning tree). Each of the approaches used enables discovering different aspects of the mutual relatedness between the cultivars. (v) phenotype associations to map information of vine utilization on the SNP landscapes.

***Allele coding and SNP-score***

In order to further process the data, we coded the genotype of each SNP using a trinary code with ‘0’ for homozygous major alleles (AA), ‘1’ for alleles (Aa and aA), and ‘2’ for homozygous minor alleles (aa).

First, let us consider one SNP in the data set of S cases, e.g. of S=783 cultivar samples. The fractions of the three genotypes of this SNP is defined as p_ij_ = S_ij_/ S (i,j= A, a), where S_ij_ is the number of SNPs with the respective allele in the data set. The minor allele frequency of a SNP is MAF= p_aa_ + 1/2 (p_aA_ + p_Aa_) with p_aa_ < p_AA_. With the SNP-code introduced above one find for its mean value averaged over all cases, <SNP-code> = p_AA_ * 0 + (p_Aa_ + p_aA_) * 1 + p_aa_ *2 = 2 MAF, i.e. the mean SNP-code of a SNP equals twice its MAF. For further data processing we define a SNP-score by centralizing the SNP-code of each SNP with respect to its mean value, SNP-score = SNP-code – 2 MAF. One obtains

SNP-score = - 2 MAF for the major allelic SNPs,

SNP-score = 1 – 2 MAF for heterozygous SNPs and

SNP-score= 2 (1 – MAF) for the minor allelic SNPs.

Second, let us consider a group of n correlated SNPs in the data set and calculate the mean of their SNP-score in one selected sample. Such a group of SNPs is given, e.g. by a meta-SNP or a ‘spot-module’ as obtained below in SOM-analysis. Analogous consideration as above deliver the result for the group-averaged SNP-score,

<SNP-score>= 2 (MAF - <MAF>)

where <…> here denotes group averaging referring to one selected sample. Hence, the group-averaged SNP-score estimates the deviation of the mean MAF of the considered SNPs in a certain sample from their mean in the considered population. Accordingly, it can be understood as an excess MAF-value (eMAF), i.e.,

eMAF= <SNP-score>/2

where positive SNP-scores mean higher frequencies than in the population while negative values refer to reduced frequencies. Centralization of each feature (here the SNP-code of each SNP) with respect to its mean value averaged over all samples is applied as standard preprocessing step in SOM analysis. Features space of centralized values is more sensitive to subtle differences between samples than feature space of non-centralized values.

***SOM portrayal and spot detection***

SOM-portrayal of SNP data was performed as follows: Our SOM implementation used a ternary code with the values 0, 1 and 2 for major homozygous, heterozygous and minor heterozygous genotypes, respectively, as introduced above. Next, SNP data was mean centralized and then clustered using SOM machine learning. SOM training translates the original data matrix consisting of the allele scores of G= 10,206 SNPs collected from S= 783 cultivar accessions into a data matrix of reduced dimensionality of M= 2,025, so-called meta-SNP profiles. Hereby, the term ‘profile’ denotes the vector of eMAF score values across the cultivars. The SOM training algorithm distributes the SNPs over the M micro-clusters of meta-SNPs by minimizing the *Euclidean* distance between the SNP- profiles as a similarity measure. It ensures that SNPs with similar profiles cluster together in the same or in closely located meta-SNPs. Each meta-SNP profile can be interpreted as the mean profile averaged over all SNP profiles of the respective meta-SNP cluster. For each cultivar accession, the meta-SNP score obtained provides the excess MAF (eMAF, see above). The eMAF values of each cultivar accession are visualized by arranging them into a two-dimensional M= 45 x 45 grid and by using a red to blue color-code for maximum to minimum eMAF-values in each of the grid images. These images ‘portray’ the genetic landscape of each accession studied in units of the eMAF SNP-score. Mean SNP-SOM portraits of cultivars from the same geographic regions, genetic components and phenotypes were obtained by averaging the meta-SNP values of the respective individual SNP-portraits. The self-organizing properties of the SOM algorithm generates red spot-like regions referring to correlated SNP-profiles showing high eMAF-values in the respective accession portraits. We used segmentation algorithms developed previously (1, 2) to extract so-called spot-clusters from these (red) regions. Each of these spot-clusters includes hundreds of SNPs.

References

1. Wirth H, von Bergen M, Binder H. Mining SOM expression portraits: Feature selection and integrating concepts of molecular function BioData Mining. 2012;5:18.

2. Wirth H, Löffler M, von Bergen M, Binder H. Expression cartography of human tissues using self organizing maps. BMC Bioinformatics. 2011;12(1):306.
